# Supplementary material for: EVI2B Is a Prognostic Biomarker and Is Correlated with Monocyte and Macrophage Infiltration in Osteosarcoma Based on an Integrative Analysis
Source: Biomolecules. 2023 Feb 8;13(2):327. doi: 10.3390/biom13020327 (PMC9953216; doi:10.3390/biom13020327)
Supplement: Supplementary file 1 [file biomolecules-13-00327-s001.zip › biomolecules-2070340-supplementary.pdf]

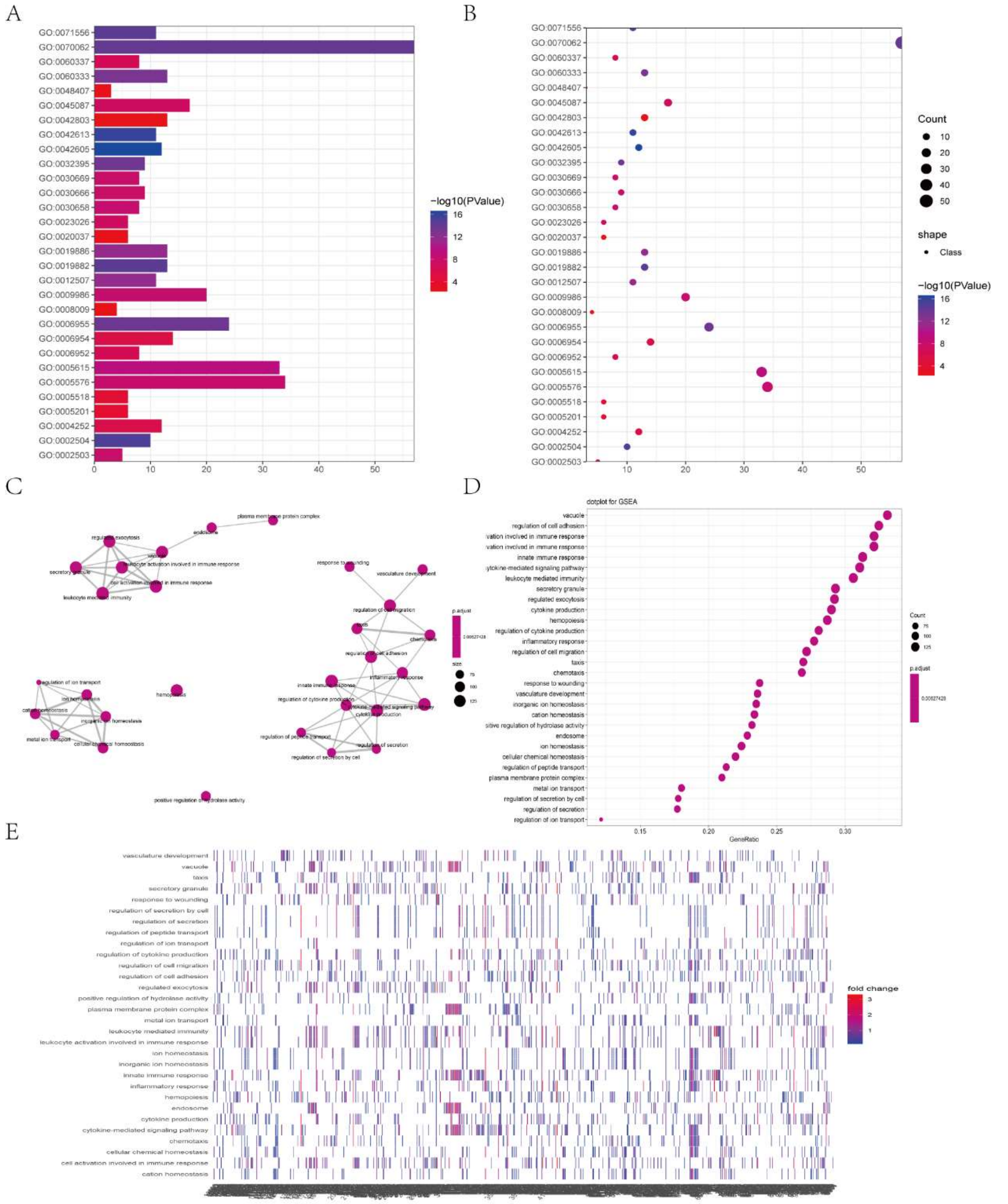

Figure S1 GO enrichment analysis (A)Bar chart of GO enrichment analysis. The length represents gene enrichment amount and color represents significance. (B) Bubble diagram of GO enrichment analysis. The bubble size represents gene enrichment number and color represents significance. (C-E) GO enrichment analysis was performed on all the DEGs.Top30 enrichment results obtained by GSEA method were respectively presented in the form of GO enrichment graph (C), bubble graph (D) and heat map (E).

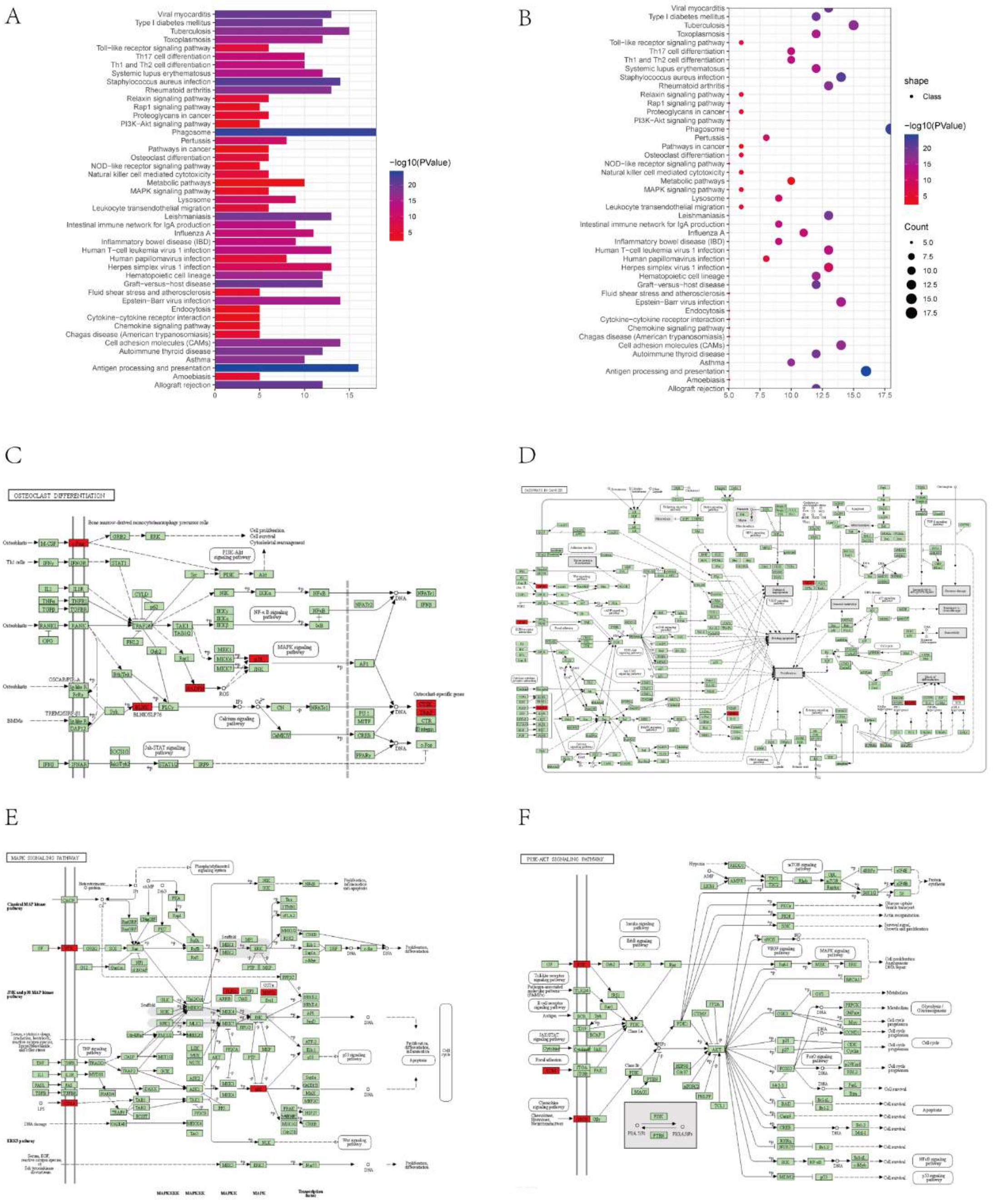

Figure S2 KEGG enrichment Analysis (A) Bar chart of KEGG enrichment analysis of KOBAS database. The length represents the number of gene enrichment, and the color increases from blue to red with increasing significance. (B) The bubble diagram of KEGG enrichment analysis in KOBAS database. The bubble size represents the number of gene enrichment, and the color increases from blue to red with increasing significance. (C) Osteoclast differentiation pathway. (D) Cancer-related pathway. (E)MAPK signaling pathway. (F) PI3K-Akt signaling pathway.

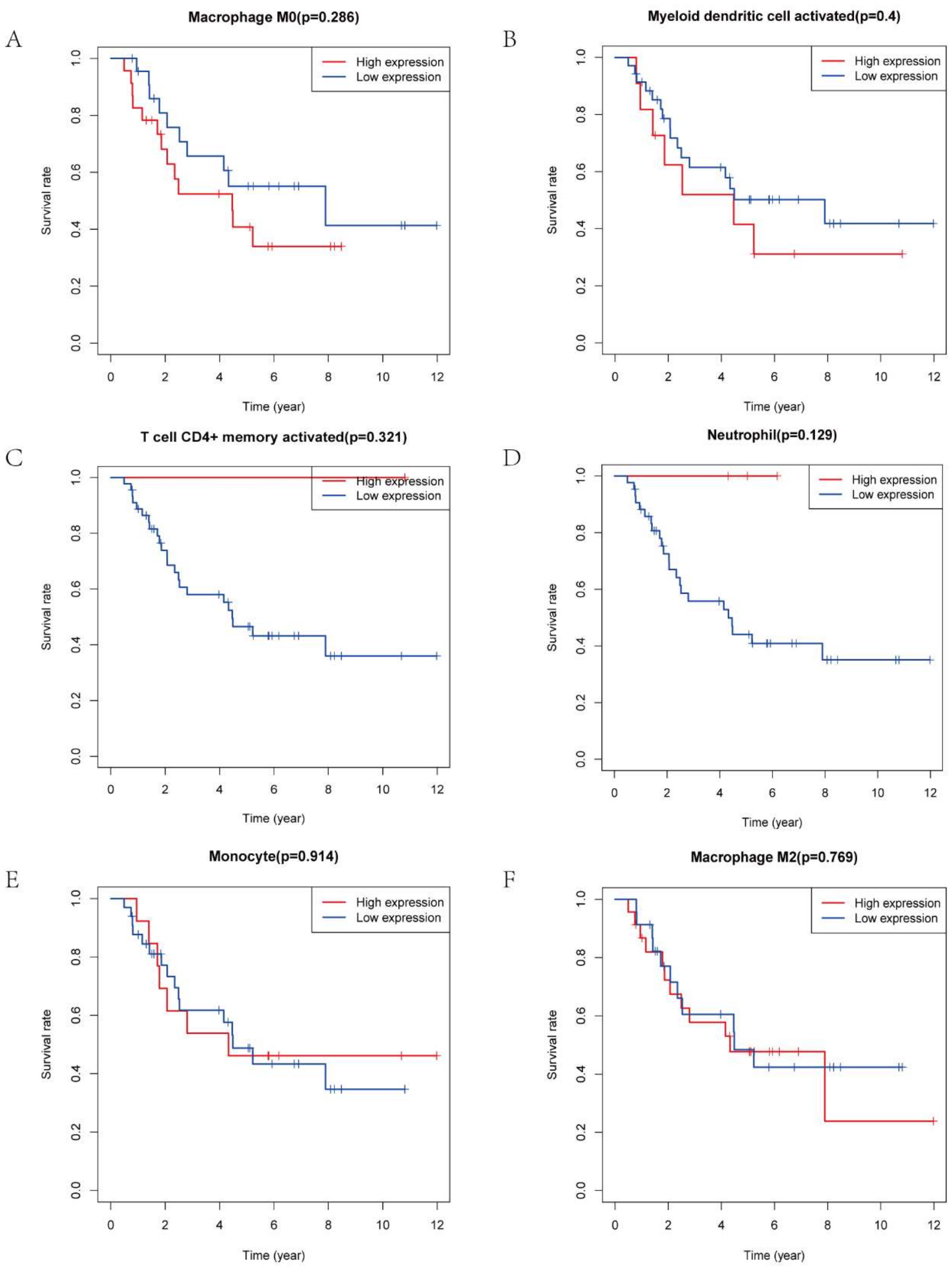

Figure S3 Survival analysis of immune cells in low EVI2B expression group. (A) Survival analysis of Macrophage M0. (B) Survival analysis of Dendritic cell activated. (C) Survival analysis of T cell CD4+ memory activated. (D) Survival analysis of Neutrophil. (E) Survival analysis of Monocyte. (F) Survival analysis of Macrophage M2.

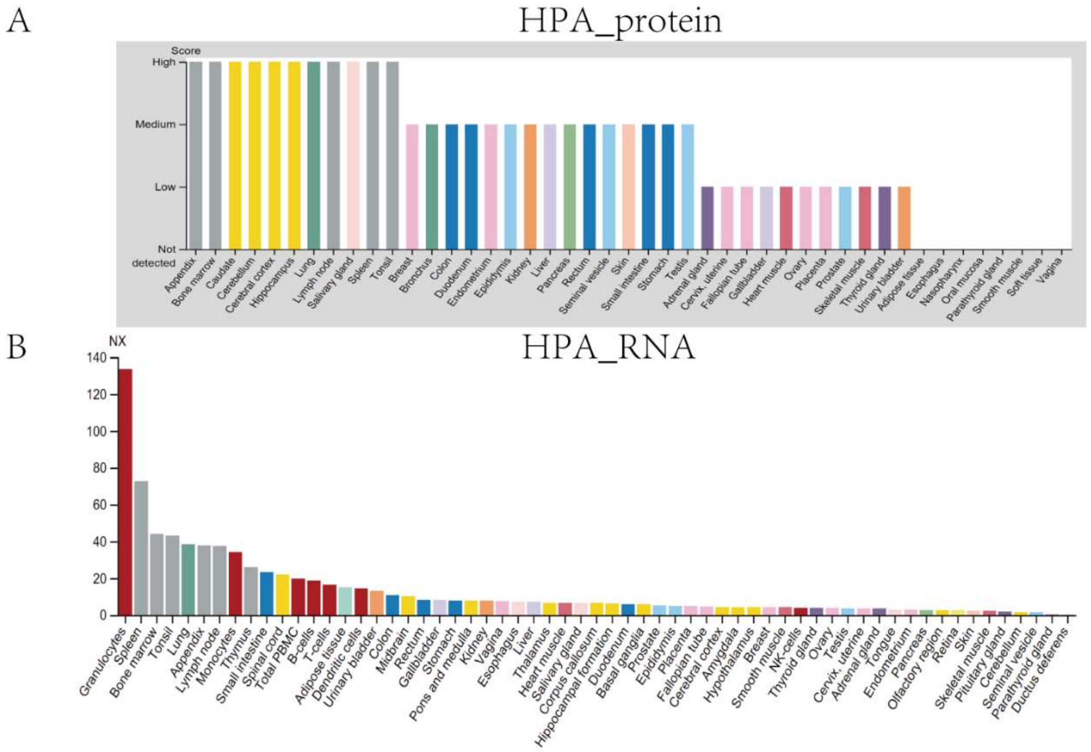

Figure S4 RNA and protein expression of EVI2B in different tissues (A) Protein expression level of EVI2B was analyzed in HPA database. (B) RNA expression level of EVI2B was analyzed in HPA database.

Table S1. The top ten GO enrichment item of DEGs in GSE42352

Category	Term	Count	PValue
BP	GO:0002504~antigen processing and presentation of peptide or polysaccharide antigen via MHC class II	10	7.64E-16
BP	GO:0019882~antigen processing and presentation	13	3.14E-15
BP	GO:0006955~immune response	24	1.99E-14
BP	GO:0060333~interferon-gamma-mediated signaling pathway	13	8.21E-14
BP	GO:0019886~antigen processing and presentation of exogenous peptide antigen via MHC class II	13	2.05E-12
BP	GO:0002503~peptide antigen assembly with MHC class II protein complex	5	1.19E-08
BP	GO:0045087~innate immune response	17	6.23E-08
BP	GO:0060337~type I interferon signaling pathway	8	3.34E-07
BP	GO:0006952~defense response	8	4.60E-07
BP	GO:0006954~inflammatory response	14	2.89E-06
CC	GO:0042613~MHC class II protein complex	11	1.04E-16
CC	GO:0071556~integral component of lumenal side of endoplasmic reticulum membrane	11	3.08E-15
CC	GO:0070062~extracellular exosome	57	7.59E-15
CC	GO:0012507~ER to Golgi transport vesicle membrane	11	2.15E-12
CC	GO:0005615~extracellular space	33	4.03E-10
CC	GO:0009986~cell surface	20	5.98E-09
CC	GO:0030658~transport vesicle membrane	8	6.74E-09
CC	GO:0005576~extracellular region	34	8.38E-09
CC	GO:0030669~clathrin-coated endocytic vesicle membrane	8	1.18E-08
CC	GO:0030666~endocytic vesicle membrane	9	1.71E-08
MF	GO:0042605~peptide antigen binding	12	2.33E-17
MF	GO:0032395~MHC class II receptor activity	9	2.76E-14
MF	GO:0023026~MHC class II protein complex binding	6	6.29E-08
MF	GO:0004252~serine-type endopeptidase activity	12	1.73E-06
MF	GO:0005518~collagen binding	6	6.16E-05

MF	GO:0005201~extracellular matrix structural constituent	6	1.05E-04
MF	GO:0048407~platelet-derived growth factor binding	3	0.0026
MF	GO:0020037~heme binding	6	0.0028
MF	GO:0008009~chemokine activity	4	0.0049
MF	GO:0042803~protein homodimerization activity	13	0.0050

Table S2. KEGG enrichment items through KOBAS database of DEGs in GSE42352

Term	ID	Count	P-Value	Corrected P-Value
Phagosome	hsa04145	18	4.89E-24	4.06E-22
Antigen processing and presentation	hsa04612	16	4.87E-25	8.09E-23
Tuberculosis	hsa05152	15	4.06E-18	6.12E-17
Staphylococcus aureus infection	hsa05150	14	5.37E-22	2.97E-20
Cell adhesion molecules (CAMs)	hsa04514	14	9.49E-18	1.21E-16
Epstein-Barr virus infection	hsa05169	14	6.14E-16	6.79E-15
Viral myocarditis	hsa05416	13	9.16E-21	3.04E-19
Leishmaniasis	hsa05140	13	1.05E-19	2.19E-18
Rheumatoid arthritis	hsa05323	13	1.21E-18	2.02E-17
Human T-cell leukemia virus 1 infection	hsa05166	13	4.83E-14	4.22E-13
Herpes simplex virus 1 infection	hsa05168	13	7.92E-10	5.06E-09
Allograft rejection	hsa05330	12	5.74E-21	2.38E-19
Graft-versus-host disease	hsa05332	12	1.25E-20	3.47E-19
Type I diabetes mellitus	hsa04940	12	2.05E-20	4.87E-19
Autoimmune thyroid disease	hsa05320	12	1.84E-19	3.40E-18
Hematopoietic cell lineage	hsa04640	12	1.30E-16	1.55E-15
Toxoplasmosis	hsa05145	12	7.08E-16	7.34E-15
Systemic lupus erythematosus	hsa05322	12	4.35E-15	4.24E-14
Influenza A	hsa05164	11	1.56E-12	1.13E-11
Asthma	hsa05310	10	9.49E-18	1.21E-16
Th1 and Th2 cell differentiation	hsa04658	10	1.58E-13	1.31E-12
Th17 cell differentiation	hsa04659	10	6.40E-13	4.83E-12
Metabolic pathways	hsa01100	10	0.004244962	0.010676722
Intestinal immune network for IgA production	hsa04672	9	3.60E-14	3.32E-13
Inflammatory bowel disease (IBD)	hsa05321	9	3.61E-13	2.85E-12
Lysosome	hsa04142	9	7.31E-11	4.85E-10
Pertussis	hsa05133	8	5.74E-11	3.97E-10
Human papillomavirus infection	hsa05165	8	2.96E-06	1.54E-05

Toll-like receptor signaling pathway	hsa04620	6	4.43E-07	2.73E-06
Leukocyte transendothelial migration	hsa04670	6	6.71E-07	3.98E-06

Table S3. Differentially expressed lncRNAs in Target-OS samples.

id	logFC	AveExpr	t	P.Value	adj.P.Val	B
RP11-750H9.5	-3.93135	4.079809	-8.51083	1.82E-13	2.65E-09	18.94929
RP11-219E7.1	-3.73355	4.07632	-5.29612	7.07E-07	0.003098	5.543843
RP11-389C8.2	-1.92275	2.969269	-4.94671	3.06E-06	0.006951	4.245193
RP11-218C14.8	-1.89195	2.083094	-4.861	4.36E-06	0.007056	3.934228
RP11-702B10.2	-6.27235	4.859972	-4.76023	6.56E-06	0.008428	3.572735
RP11-589N15.2	-5.39793	6.781961	-4.73127	7.37E-06	0.008428	3.469656
GAPLINC	-5.26976	11.63898	-4.72633	7.51E-06	0.008428	3.452128
RP5-899E9.1	-4.56207	3.94466	-4.70555	8.17E-06	0.008445	3.378425
AC079767.4	-1.60142	1.459407	-4.67401	9.27E-06	0.008445	3.267001
RP11-802E16.3	-14.131	16.3885	-4.57051	1.40E-05	0.011117	2.904509
CARD8-AS1	-1.43683	3.335462	-4.56135	1.45E-05	0.011117	2.872668
RP11-807H22.7	-2.43514	2.522574	-4.40773	2.64E-05	0.016022	2.344731
HLA-DQB1-AS1	-1.24959	1.173702	-4.26106	4.62E-05	0.024173	1.851652
RP11-681H18.2	-13.5191	26.78004	-4.25976	4.64E-05	0.024173	1.847324
RP11-99E15.2	-1.05733	1.171499	-4.24527	4.90E-05	0.024654	1.799203
CH17-340M24.3	-4.10538	7.251198	-4.23602	5.08E-05	0.024678	1.76855
THCAT158	-9.18147	9.273161	-4.21777	5.44E-05	0.02558	1.708209
RP11-132A1.4	2.836214	2.044146	4.181032	6.24E-05	0.027578	1.587273
ZBED5-AS1	-5.48064	10.2802	-4.13143	7.51E-05	0.030787	1.425121
LINC00622	-2.75604	3.570492	-4.12732	7.62E-05	0.030787	1.411734
RP11-344B5.2	-1.61499	2.457995	-4.06713	9.52E-05	0.03426	1.216874
RP11-73M18.8	-8.73536	22.34209	-4.05988	9.78E-05	0.03426	1.193541
RP11-320M16.2	-3.41895	3.167094	-4.00263	1.21E-04	0.040316	1.010277
AC092652.1	-2.13451	1.737889	-3.99386	1.24E-04	0.040316	0.982351
RP11-314B1.2	-1.64748	1.271104	-3.97008	1.36E-04	0.042092	0.906876
RP11-472N13.3	-1.08577	1.54673	-3.89659	1.77E-04	0.048632	0.67565
